# The reimagination of school-based physical activity research in the COVID-19 era

**DOI:** 10.1371/journal.pmed.1003267

**Published:** 2020-08-28

**Authors:** Deirdre M. Harrington, Michelle O’Reilly

**Affiliations:** 1 Lecturer in Physical Activity, Sedentary Behaviour and Health, Diabetes Research Centre, University of Leicester, Leicester, United Kingdom; 2 Associate Professor of Communication in Mental Health, School of Media, Communication and Sociology and School of Psychology, University of Leicester, Leicester, United Kingdom; 3 Research Consultant, Leicestershire Partnership NHS Trust; Leicester, United Kingdom

## Abstract

Deirdre Harrington and Michelle O'Reilly discuss the article "Effectiveness and cost-effectiveness of the GoActive intervention to increase physical activity among UK adolescents: A cluster randomised controlled trial" by Kirsten Corder and colleagues.

The coronavirus disease 2019 (COVID-19) pandemic has illuminated the chasm of societal inequality and vulnerability within the United Kingdom concurrently highlighted by the February 2020 Marmot Review [1]. These inequalities and vulnerabilities have been omnipresent in other public health crises, such as child mental health [2], childhood obesity levels [3], and, of most relevance to this piece, physical inactivity [4]. Rather than addressing the underlying inequalities driving these crises, governments have placed schools on the frontline of delivering solutions.

Researchers also recognise the attractiveness of the school setting for applying research-driven solutions to childhood physical inactivity because the majority of young people can be easily reached. Evaluation of school-based physical activity programmes using the rigid cluster randomised controlled trial (RCT) design aim to contribute high-quality evidence on approaches to tackling physical inactivity. The National Institute for Health Research (NIHR) in the UK is one funder that has invested significant funds in large school-based cluster RCTs (see the online NIHR Journals Library for examples). These trials overcame methodological weaknesses identified in previous systematic reviews [5] by having a robust design, adequate power, using objective behaviour measures, having longer follow-up, and evaluated contextually appropriate programmes with, when possible, a theory and feasibility evidence base.

The Corder and colleagues paper in this *PLOS Medicine* special issue [6] is the latest example of such a trial. The authors report on the effectiveness and cost-effectiveness of a theory-driven, feasibility-tested physical activity promotion programme for 12 to 14 year olds in 16 UK secondary schools. The GoActive programme saw mentors and peer leaders encouraging pupils to try novel physical activities inside and outside of school time. The programme ran over 12 weeks; 6 weeks were supported by external facilitators from the community before this support was phased out. Despite the potential, the GoActive programme was not effective at preventing declines in physical activity in the regional sample of UK adolescents. The difference in objectively measured moderate- to vigorous-intensity physical activity was −1.9 (confidence interval: −5.5 to 1.7) minutes/day at 10 month post intervention follow-up. At a cost of £13 per pupil, it was also deemed not to be cost effective. In line with a recent review of trial implementation [7], issues with fidelity in programme delivery (between 12% and 64% of students across schools attended a GoActive session and less than 50% engaged with the accompanying website) are posited to be responsible.

Despite the methodological quality of this and previous trials, effectiveness (through the narrow definition of positively affecting the primary outcome) has been limited. Little new knowledge of what works, or clear results that have led to policy or provision change, has been produced from these trials. It is clear that doing more of the same will not lead to the creation of new knowledge relevant to stakeholders. This is particularly pertinent because the Corder publication coincides with the crest of the COVID-19 pandemic—a global issue that means researchers and funders are likely to be forced to reassess their priorities and approach.

## Will schools be seen as the solution to the post-COVID-19 ‘problems’?

The general area of ‘health, sport and physical education’ in schools is commonly used as a vehicle to address public health or societal ‘problems’ [8]. UK Prime Minister Boris Johnson is said to be primed ‘to launch a war on fat’ [9], and we expect schools to remain an attractive location for any efforts. Schools may not be able to devote ‘time and energy’ into solving whatever the latest crisis is as ‘they are neither expert nor likely to have an impact’ [10], but teachers do have a desire for expertise [8]. However, after 10 years of austerity, teachers are at their limit for what they can deliver outside of the curriculum. Corder and colleagues highlight issues with teacher burden (‘time pressures’) and future sustainability of the GoActive delivery model. The team trained peer mentors and had local authority-funded health trainers facilitate half of the delivery that ultimately negatively affected fidelity [6].

## What are the implications of social distancing for data collection in schools?

The research world has quickly mobilised and repurposed studies to tackle the COVID-19 pandemic including research on how health behaviours and outcomes, such as physical activity, have changed. School-based physical activity research too will likely change. The need for social distancing and enhanced personal hygiene practices would pose practical challenges if we imagine planning the GoActive study methods in the COVID-19 era.

The Corder trial employed a cluster RCT design that required large amounts of data collected during a narrow time frame to fit in with the academic calendar (because intervention delivery is usually anchored to term times). This is done by teams of research staff travelling together, sharing equipment, and co-operating in settings the researchers often have little control over. Collection of outcome data such as waist circumference requires close proximity between research staff and participants. The rapid deployment, use, and collection of body-worn physical activity measurement devices would require careful hygiene procedures. Research staff would facilitate large groups of pupil participants completing extensive questionnaire booklets in packed classrooms. The willingness to engage in these data collection methods will largely be down to the researchers’ own personal assessments of risk and that of the school.

## The ‘new normal’ is not clear for researchers or schools

As the world still reels from the effect of the COVID-19 pandemic, there is an impending threat of triple inertia: that funders may stop funding school-based physical activity research, that academics may flounder under the weight of evidence lacking effectiveness, and that stakeholders will have new priorities resulting in limited physical activity research opportunities with schools.

The stark and transparent light of COVID-19 provides the UK government a real chance to tackle health inequality and to reinstate local government public health and education funding that schools rely on. This would provide schools with adequate financial and human resources to develop bespoke physical activity programmes. These practice-driven programmes are more likely to suit the needs and preferences of the school’s own pupils and account for and listen to adolescents’ perspectives as well as other key stakeholders. This would open up the opportunity for pragmatic and flexible evaluation of these practice-led programmes in schools allowing researchers to move away from RCTs and towards more realist [11] methodologies. Now more than ever researchers need to be more innovative and interdisciplinary in research endeavours as we start to chart the ‘new normal’ for school-based physical activity research.

## Abbreviations

COVID-19: coronavirus disease 2019
RCT: randomised controlled trial
